# Design of the Dual Stone Locating System on an Extracorporeal Shock Wave Lithotriptor

**DOI:** 10.3390/s130101319

**Published:** 2013-01-21

**Authors:** Yong-Ren Pu, Ioannis Manousakas, Shen-Min Liang, Chien-Chen Chang

**Affiliations:** 1 Department of Occupational Safety and Health, Chang Jung Christian University, Tainan City 71101, Taiwan; 2 Department of Biomedical Engineering, I-Shou University, Kaohsiung City 84001, Taiwan; E-Mail: i.manousakas@ieee.org; 3 Department of Computer Application Engineering, Far East University, Tainan City 74448, Taiwan; E-Mail: liangsm@cc.feu.edu.tw; 4 Department of Urology, National Cheng Kung University Hospital, Tainan City, 70403, Taiwan; E-Mail: fred@mail.ncku.edu.tw

**Keywords:** X-ray, ultrasound, image processing, Extracorporeal Shock Wave Lithotriptor, stone location, stone tracking

## Abstract

Extracorporeal Shock Wave Lithotriptors are very popular for the treatment of urinary stones all over the world. They depend basically upon either X-ray fluoroscopy or ultrasound scans to detect the stones before therapy begins. To increase the effectiveness of treatment this study took advantage of both X-ray and ultrasound to develop a dual stone locating system with image processing modules. Its functions include the initial stone locating mode with stone detection by fluorescent images and the follow-up automatic stone tracking mode made by constant ultrasound scanning. The authors have integrated both apparatus and present the operating principles for both modes. The system used two *in vitro* experiments to justify its abilities of stone location in all procedures.

## 1. Introduction

Extracorporeal Shock Wave Lithotriptors (ESWLs) have been widely utilized in the urology departments of hospitals all over the world. Their use in noninvasive treatment [1] has gained credit from the majority of doctors and patients in urology. Most of the currently operational ESWLs locate stones (calculi) manually with a C-arm (X-ray) or ultrasound (US) scanner. When the stone location is done, the lithotriptor thereafter strikes with thousands of shock waves focusing at a spatially fixed focal area (F2) where the stone presumably exists and is subsequently shattered into pieces. After treatment, the patient excretes those fragments as a matter of course during the following months [2].

The prognoses by ESWLs vary in different studies. In 2010 a large-scale research studied more than 8,500 patients and concluded that the overall stone free rates using an electromagnetic and an electrohydraulic lithotriptors were 61.1% and 64.5%, respectively [3]. A more recent study reported that the stone-free rate after 3 months was 69.4% after assessing the 3-year treatment results [4]. The above stone-free rates of the traditional lithotriptors are no more than 70%. It is because the stone moves due to the patient's respiration which causes organs to travel, sometimes by as much as 7 cm [5]. An *in vitro* study showed that the percentage of SWs hitting the stone were 70% and 39% as demonstrated by a model stone driven by a motorized positioner undergoing 10 and 18 mm of motion, respectively [6]. Those missed shots delivering the compressive and tensile forces were consequently directed at the surrounding tissues and caused cavitation around the cells, which produces renal trauma side effects [7].

The drawbacks of the existing ESWLs include several aspects. First of all, the stone location procedure is tedious and needs a proficient operator to shorten the fluorescence exposure time. Secondly, a urinary stone moves due to the patient's respiration. Shock waves restricted to a fixed point not only reduce the effectiveness of the treatment, but may also damage nearby tissues. Finally, the operator needs to check the stone's location and reactivate the X-ray fluoroscopy so often that the patient is exposed to even higher doses of radiation. There were some efforts to increase the coincidence ratio between the stone and F2. An Anti-misshot Control Device (AMCD) has been installed in a piezoelectric lithotriptor to prevent misshots to renal tissue. It functions in such a manner that shock waves were generated only when the US echo of a stone was in the focal region [8]. Another software called Echotrack was also developed to calculate the average position of the stone in US images. It automatically adjusted the SW generator to coincide with the stone's location once every certain amount of time [9]. A genuine automatic stone tracking system was developed by the authors' research team and functioned to use US to sense the stone and servo motors to track it in real time. The system could reduce by more than half the number of SWs needed [10,11].

This study takes advantage of both X-ray and US to develop a dual stone locating system on a domestic ESWL here in Taiwan. It is capable of initially locating a urinary stone and then tracking it down automatically. The work includes the image processing on both pieces of equipment, the derivations of stone location equations, the programming of the control panel, and the integration of mechatronic components. It is noted that, in fact, some of the technologies developed in this study are not new. Few of them are even in clinical use already. Our job is to accommodate the frameworks of an existing lithotriptor and integrate these technologies for the new model to achieve our design goals.

## 2. Methods

### 2.1. System Framework

**Figure 1.**
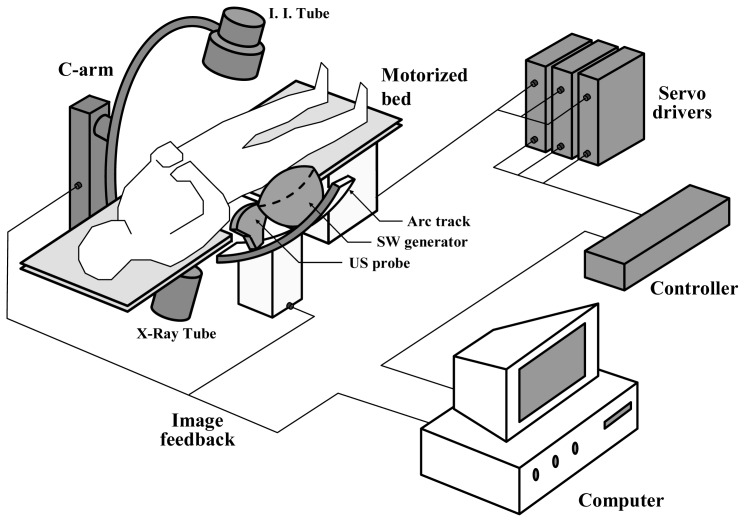
The framework of the dual stone locating system.

We integrate a C-arm, an US probe holder, a SW generator and servo mechanisms to enable the system to locate and track the stone. The framework of the system is shown in Figure 1. The SW generator and the US probe sit on an arc track whose center coincides with F2 and resides in the US scan plane. The system is equipped with servo mechanisms which make both stones and focal zones meet each other quickly and accurately. This can be done by actuating either the arc track or the bed. In our design it is the bed that is chosen to be motorized by servo motors. On the other side, a C-arm is installed next to the lithotriptor. To detect the stone inside a patient, two X-ray images are taken at different roll angles. The operator, successively, localize and mark the stone on the X-ray screen twice. The software immediately processes both marks' coordinates to calculate the stone's position from F2 in three dimensions. The computer subsequently commands the controller to actuate the servo system which moves the bed in such a way that the stone arrives at F2. The US probe then extends and takes over the procedure from there.

### 2.2. Initial Stone Location by X-Ray

**Figure 2.**
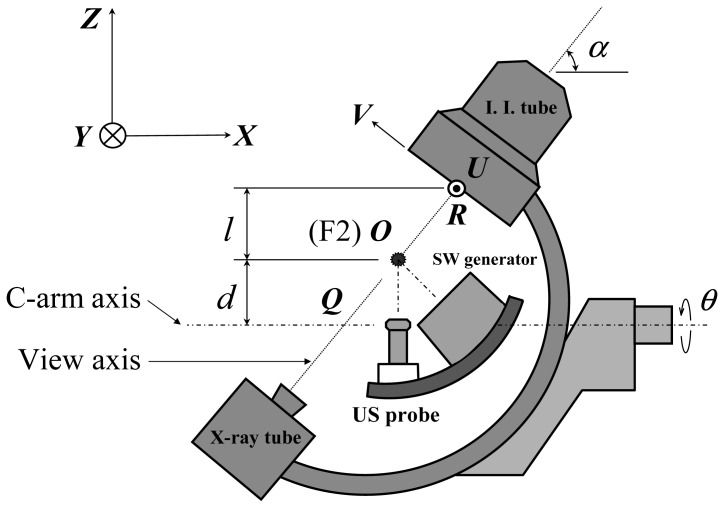
The geometry of the dual stone location apparatus.

In the initial locating mode, X-rays are used to detect the stone. We need to derive the stone's spatial coordinates from the fluorescence images. The more general geometric relations of the C-arm and the apparatus on the arc track are shown in Figure 2. For the C-arm, *α* denotes the pitch angle and *θ* the roll angle which rotates around the C-arm axis. When the C-arm is in the upright position (θ = 0°), the view axis of X-ray penetrates F2. The spatial 3D coordinate system is ***OXYZ*** where ***O*** is the origin. The local 2D coordinate system for the fluorescent image is ***RUV*** where ***R*** is the entry point of the view axis to the image intensifier tube (I. I. tube) and appears at the center of the X-ray image. Note that ***OXYZ*** appears to be the lithotriptor's space and ***RUV*** represents the plane shown in the X ray monitor, where all of the coordinate vectors are perpendicular. ***Q*** is the intersection of the view axis and the C-arm axis.

**Figure 3.**
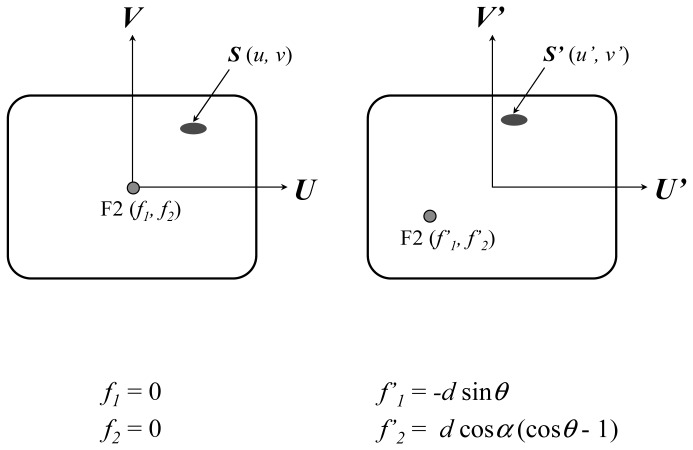
The schematic diagrams of the fluorescent images. On the left, the C-arm is upright; and, on the right, the C-arm rolls at an angle *θ*.

When the C-arm rolls at a certain degree, *θ* (***U***, ***V***) becomes (***U′***, ***V′***) and F2 no longer resides at the view axis. The schematic diagrams of two fluorescent images with different roll angles are shown in Figure 3 where F2 will be in different positions. If the stone appears in both X ray images, they need to be marked manually on the screen where they are denoted by ***S*** and ***S′*** with local coordinates (*u*, *v*) and (*u′*, *v′*), respectively.

Once the local coordinates of ***S*** and ***S′*** on the fluorescent images are determined by image processing, the distance of the stone from F2 in spatial coordinates can be derived as follows:

(1) $$x=\frac{u\cos\alpha \cos\theta -(u'+d\sin\theta )\cos\alpha -v\sin\theta }{\sin\alpha \sin\theta }$$

(2) y=−u

(3) $$z=\frac{u\cos\theta -u'-d\sin\theta }{\sin\theta }$$

The stone can subsequently be positioned by driving the bed with motors, since (*x, y, z*) is in the ***OXYZ*** coordinates space where all motors reside. The result is verified by reactivating the X-ray to check whether ***S*** (stone) is at the center. The system will then go into the stone tracking mode.

### 2.3. Stone Tracking by Ultrasound

**Figure 4.**
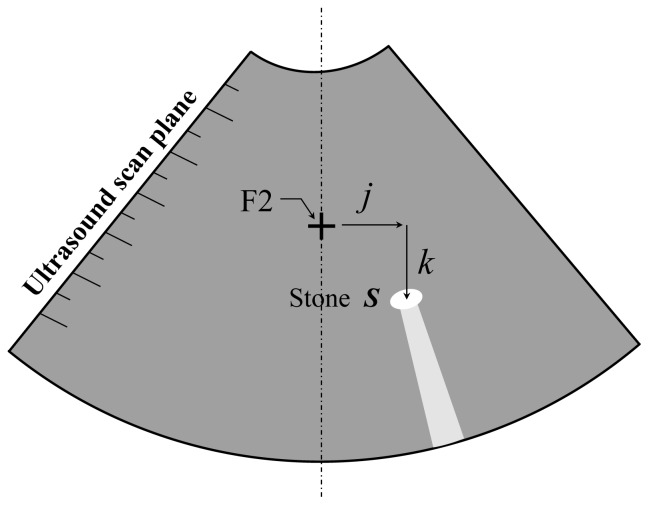
The schematic diagram of the stone deviation in the US images.

As the stone is around F2 and within the US scan plane, it will be seen in the US image with an obvious acoustic shadow as schematically shown in Figure 4. The stone deviation from F2 can also be determined by two image processing techniques [10]: first, the stone is centered in a region of interest (ROI) and detected by gray level histogram entropy [12]; and next, the frame matching technique is used to identify the deforming stone image as follows. A simple binary matching procedure between successive frames is implemented. A figure of merit (*FOM*) is incorporated to quantify the match. The figure of merit consists of the count of the pixels set to “1” in both the ROIs in the two frames:

(4) $$\text{FOM}=\sum_{\text{for}\;x,y\;\text{and}\;i,j\;\text{where}\;P\;\text{and}\;C\;\text{overlap}\;}\;W\cdot [P_{x,y}(\text{AND})C_{i,j}]$$

where *P* and *C* are the ROIs in the previous and current frames, respectively, and *W* is a normalization factor. The algorithm suggests that the highest *FOM* corresponds to a match of the largest region between both frames in which there is a relative offset. Therefore, the stone in the current frame is found to be at the position in the previous frame plus a displacement as much as the offset. Once the procedure is done, a new ROI of the current frame can be determined and keep locking on the stone.

**Figure 5.**
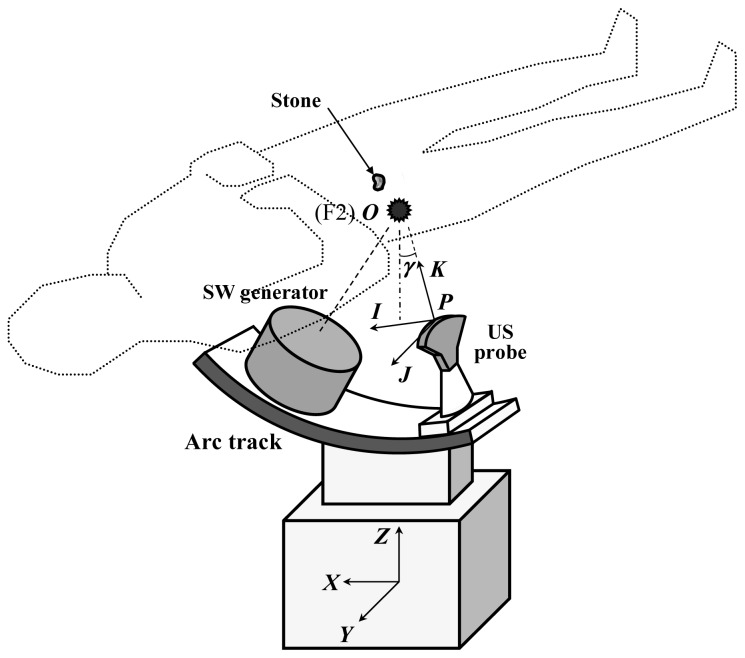
The coordinate system of the US probe for stone tracking.

In order to successfully track the stone, we need to realize the construction of the arc track as shown in Figure 5. For convenience of treatment, the operator might swing the arc track about F2 until the US scan plane has an angle *γ* from the vertical. ***O*** and ***P*** denote F2 and the tip of the US probe, respectively. The spatial coordinate system is ***OXYZ***. The local coordinate system is ***PIJK***, part of which is reflected in the US image in Figure 4. This means that *PJK* represents the plane shown in the US monitor. Since the stone deviation is found to be ***OS*** = *j****J*** + *k****K*** in local coordinates, we rewrite:

(5) OS=ksinγX+vY+kcosγZ

in spatial coordinates. At any instant, the motorized bed can compensate this deviation by servo mechanisms to bring the drifting stone back to the focal zone.

Using US scanning means that the stone has to be confined within the scan plane. It is hard to tell which way the stone goes when it disappears. Fortunately, due to respiration, the stone is moving back and forth mostly in the longitudinal direction rather than in the sagittal and frontal ones. It seldom escapes from the US scanning, which makes tracking the stone for a long period of time possible.

## 3. Results and Discussion

**Figure 6.**
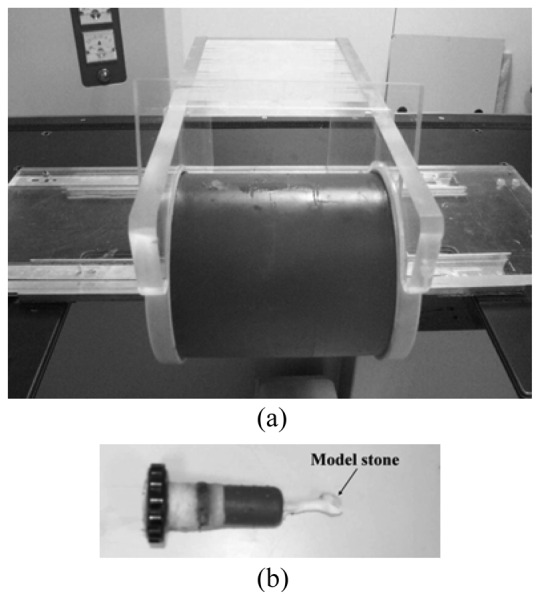
(**a**) The phantom with kidney-shaped cavity mounted on the ESWL, and (**b**) the phantom cap attached with a model stone at the tip.

We performed two *in vitro* experiments to demonstrate the ability of the dual stone locating system. The first was the initial stone location inside a phantom (LTK-5, ATS Lab., Inc., Bridgeport, CT, USA, see Figure 6). A model stone attached to the tip of the phantom cap was plugged inside the kidney-shaped cavity of the phantom. The phantom was mounted on the ESWL bed and placed inside the exposure range of the X-ray.

**Figure 7.**
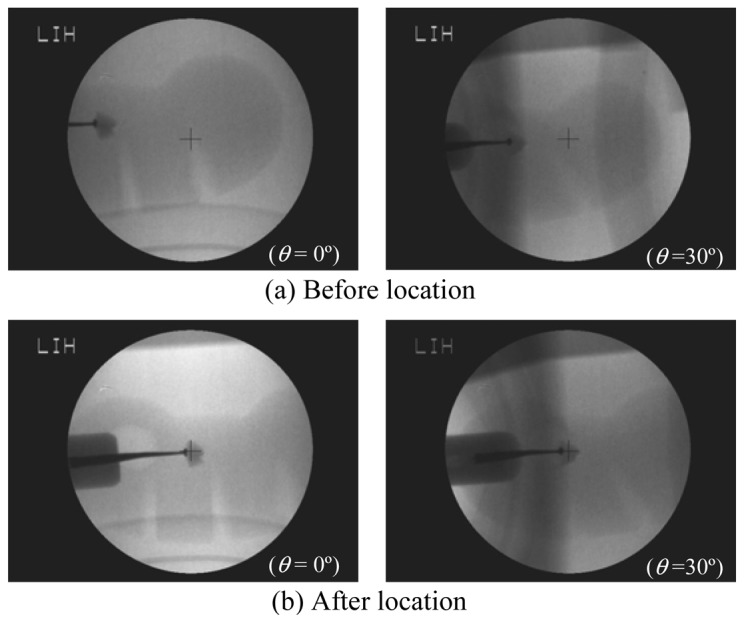
The fluorescent images of the phantom (**a**) before stone location, and (**b**) after stone location.

Since there was not any obstructing factor along the view axis, no pitch angle was needed (*α* = 0°). Two consecutive fluorescent images (*θ* = 0° and *θ* = 30°) were taken and displayed on the monitor to let the operator point at the stone. According to Equations (1)–(3), the system actuated the servo mechanisms to move the bed briefly. Afterward, another two consecutive fluorescent images with the same angles were taken to verify the accuracy of the stone location, as shown in Figure 7. As one can see, the initial stone location mode did successively bring the modal stone to F2 (cross mark).

**Figure 8.**
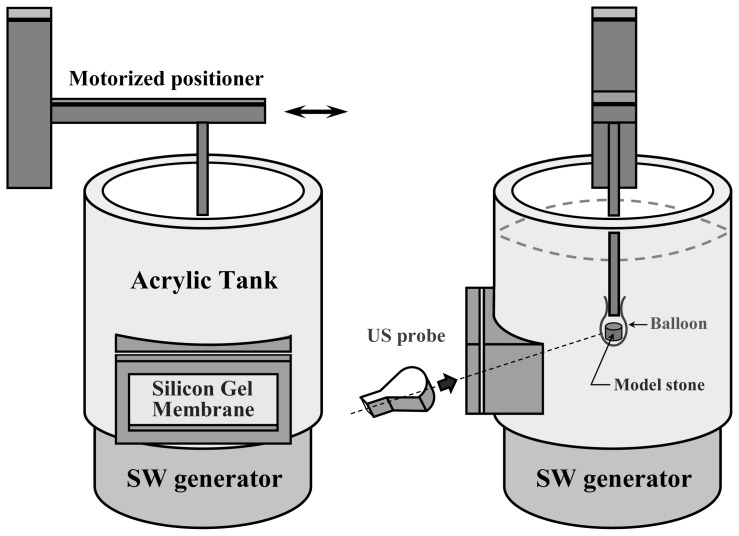
The front (left) and side (right) views of the devices for the *in vitro* automatic stone tracking experiment.

The second experiment was to demonstrate the automatic stone tracking ability as the system switched to the stone tracking mode. To simulate the stone moving inside a patient, we customized a set of devices to let the US be able to scan a model stone. Figure 8 shows an acrylic tank mounted on the SW generator that included a side window. The US probe could make contact with the silicone gel membrane fixed to the window frames and scan inside the water tank. A motorized positioner immersed a stick whose tip was fastened with a water-filled balloon containing a cylindrical model stone (6 × 6 mm, diameter × height.) A previously recorded trajectory of a kidney stone was introduced to the positioner that moved the stone to simulate a situation perturbed by respiration. When the automatic tracking mode was turned on, the software continuously processed the US images in real time (∼10 fps) and simultaneously commanded the servo mechanisms to move the bed along with the positioner. We recorded the US images all the way for tracking and non-tracking conditions, and later on analyzed their coincidence rates between the stone and F2 along the time axis.

**Figure 9.**
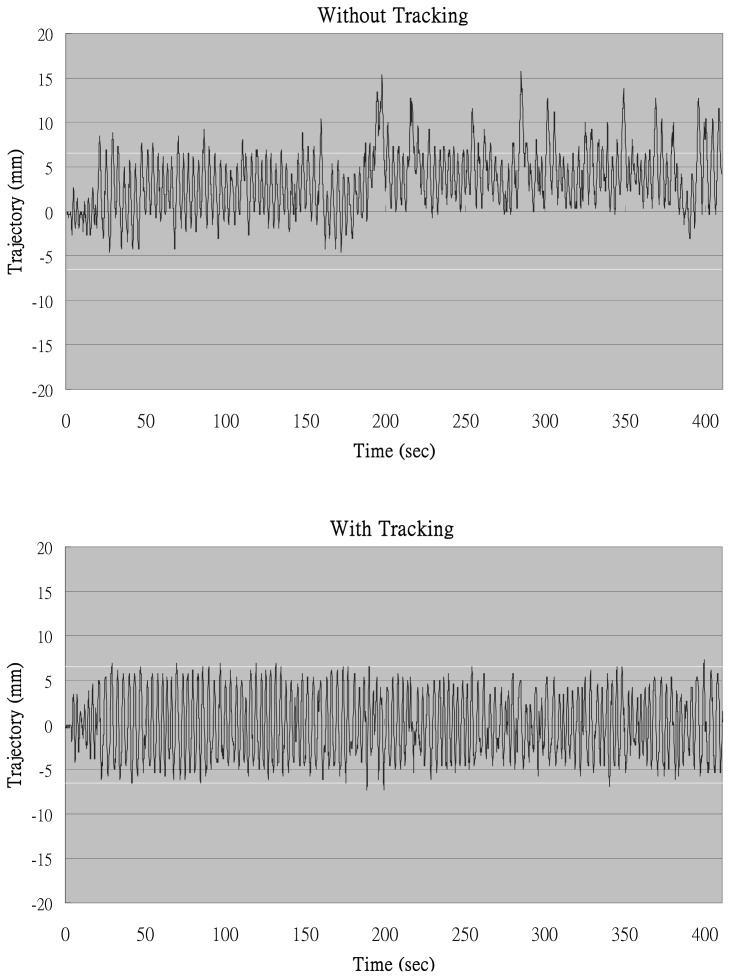
The trajectories of the model stone's center in the US images with and without tracking.

Figure 9 shows the trajectories of the stone's center in the US images with and without tracking. The horizontal dashed lines indicated that the portion of the stone is in the focal zone. This was set between ±6.5 mm where the effective range of the focal zone was ±3.5 mm and the half size of the stone was approximately 3 mm. As the stone's center went inside the dashed lines, the SW would be considered able to successfully hit the stone.

Without tracking, the patient's breathing slowly shifted the average position of the stone, which accounted for the lower coincidence rate of only 82.6%. In the automatic stone tracking mode, on the other hand, the trajectory stayed mostly inside the focal zone and reached 98.5% of the coincidence rate. This means that in this case the application of nearly 20% of the SWs could be saved by using the stone tracking function.

It is noted that, as mentioned in the last paragraph of Section 2, the system cannot track the stone if it moves out of the ultrasound scan plane. The software has to stop tracking, reposition the stone by X ray, and start tracking all over again. Although missing the stone is annoying, it seldom happens in the current *ex vivo* simulation setting without shock wave application. Besides, re-targeting on the stone does not take long and does no more harm to the patient, compared to the traditional X-ray ESWLs. If the stone breaks and its fragments distribute in various calices during treatment, as long as the ultrasound can identify one of the fragments and the contrast between the fragment and the surrounding tissue is clear, *i.e.*, it can lock on it. The tracking mode can still be activated. If continuously recognizing or tracking the stone through US is not possible, this system still can remain to target by X ray only on a fixed point without using tracking function. Just like the old way, it is better for one to target on the stone fragments piece by piece.

It needs to be mentioned that this *in vitro* experiment is used to test the tracking ability of the revised system in a simplified environment. In *ex vivo* or even *in vivo* situations the surrounding tissues of the stone might greatly change the gray level characteristics in the ultrasound images which may affect the success of recognizing and tracking the stone. These need to be assessed in the future studies. In addition, some feature in the US image, such as the anechoic shadow beneath the stone, will be implemented in the stone recognition process in future endeavors.

## 4. Conclusions

In this paper, the development of the dual stone locating system and the related principles were introduced. We have integrated both X-ray and US apparatus to locate and track stones on the ESWL. Two *in vitro* experiments were used to demonstrate the effectiveness of the system. The results showed that our design was able to correctly locate the stone through consecutive fluorescent images initially, and constantly track it down with the help of US scanning. These promising results potentially make it possible to not only shorten the treatment time and increase the efficiency of stone fragmentation, but also lessen the degree of injury to the surrounding tissues.
